# Funding research, a Chinese perspective

**DOI:** 10.1186/s13059-019-1797-x

**Published:** 2019-08-28

**Authors:** Hui Yang

**Affiliations:** Institute of Neuroscience, CAS Center for Excellence in Brain Science and Intelligence Technology, Shanghai Research Center for Brain Science and Brain-Inspired Intelligence, Chinese Academy of Sciences, Shanghai, 200031 China

## Abstract

Funding research is a challenge faced by most scientists around the world. Genome Biology has invited four scientists based in three different countries to share their own experience and opinions regarding funding, the difficulties young scientists must overcome, and how the process of securing funding can be improved. Here, Hui Yang discusses the funding opportunities open to scientists conducting research in China.

## Main text

I started my laboratory in the Chinese Academy of Science (CAS) in 2014, and I am currently a principal investigator (PI) at the Institute of Neuroscience (ION).

Starting a laboratory for a young, unestablished scientist in China might be easier relative to more developed countries. There are approximately over a thousand new PIs setting up new laboratories every year in China, and one-third of them have a chance to get awards designed for unestablished scientists, such as the ones from the China Young Scientist Program and the National Natural Science Foundation of China. In the CAS, one could also apply for the CAS Strategic Priority Research Program. In the ION, one could join the State Key Laboratory of Neuroscience and CAS Center for Excellence in Brain Science and Intelligence Technology and get good support. Some of my colleagues would apply for such funding before they decided where to start their laboratories, as the awards for unestablished scientists affects the start-up funding and the extent to which their laboratories are supported by the institutes and institutional programmes. This start-up funding helps young scientists focus on research in their early career. To be honest, I had not spent much time on writing grants in the first 3 years. For senior researchers, the competition could be tough at the state level, but they could easily get funding from local funding agencies at province or city level.

The initial funding gives young scientists the opportunity to focus on and generate impactful work, which will be mostly judged by the publications. The institutes usually value the quality of the journals in which the articles are published over the quantity of publications. It is hard to evaluate a work by citation within its first several years of publication; therefore, the impact factor of the journals is valued more than the actual citation of the article. *Cell*, *Science* and *Nature* are among the journals that are highly valued by Chinese institutions.

If scientists are having difficulty getting funding and being published, they could choose to move from big cities such as Beijing and Shanghai to smaller cities in west or central China, with some cities, such as Shenzhen, offering very competitive packages to attract talent. It usually means more personal income but a less-established core facility or platform.

In the top institutes in Beijing and Shanghai, one can easily build up a 10-person team in the first 1 or 2 years, including students, technicians and postdocs. From what I have heard, most of the Chinese institutes are similar in terms of funding and staff availability. Many talented students might be attracted to overseas laboratories, for the culture difference as well as some experience in mega- laboratories in which dozens of postdocs work together. Things have been changing recently, with Chinese laboratories becoming more appealing to talented students and postdocs.

Regarding the amount of time spent on grant writing, this mostly depends on the subject areas and the size of the laboratories, rather than the seniority of the PIs. In laboratories that are focusing on non-human primate models and techniques that are very expensive, the PIs need to spend much more time applying for funding. Although there are usually more funding options for expensive and hot projects, the amount of money available for each project is similar; therefore, applicants need to get more projects funded if they are running bigger laboratories.

The evaluation of methods of research work by Chinese institutions still has scope for improvement. Some of the institutions have realized that, in a specific subject area, there are few journals with a high impact factor, and some well-respected journals are accepted as a standard for excellent work. A more comprehensive evaluation system is needed.

